# Industrial Internet of Things for a Wirelessly Controlled Water Distribution Network

**DOI:** 10.3390/s25082348

**Published:** 2025-04-08

**Authors:** Mahmud M. Nagasa, Princy L. D. Johnson

**Affiliations:** School of Engineering, Liverpool John Moores University, Byrom Street, Liverpool L3 3AF, UK; p.johnson@ljmu.ac.uk

**Keywords:** automation, industrial internet of things (IIoT), network topology, real-time systems, scalability, trusted wireless, wide area networks (WANs), wireless networks, wireless communication protocols

## Abstract

This paper presents two innovative wireless network designs for the automation system of the Sof-Algeen water station in Zintan, addressing the challenge of connecting field instruments—such as pressure switches, solenoid valves, and differential pressure sensors—over distances of up to 4 km. Due to high costs, limited flexibility, and scalability concerns, traditional hardwired solutions are impractical for such distances. A comprehensive analysis of various Industrial Internet of Things (IIoT) network designs determined that the IEEE 802.11 standard and Phoenix Contact’s Trusted Wireless technology best meet the project’s requirements for long-distance connectivity, real-time data acquisition, system compatibility, and compliance with national telecommunications regulations. This study proposes optimal network designs using the IEEE 802.11 standard and a hybrid mesh and star network for Trusted Wireless, and evaluates these technologies based on performance, reliability, and infrastructure compatibility using simulation. The network designs were validated using the Radio Mobile tool, considering the water station’s specific terrain and wireless module parameters. The findings indicate distinct differences in structure, operation, and cost-effectiveness between the two proposed solutions, highlighting the benefits of each in achieving optimal link feasibility for robust water station automation.

## 1. Introduction

Wireless networks are revolutionizing industrial automation by offering a more flexible and cost-effective alternative to traditional wired systems, simplifying cabling and enabling easy reconfiguration of production layouts [1]. In Zintan, northwest Libya, the Sof-Algeen water station, located 30 km south, serves as the primary water source. Currently, its operations are manual and outdated, leading to issues such as tank overflows, pipeline leaks, and pump dry running, posing significant challenges to the city’s water management efforts [2].

In response to these challenges, the integration of digital technologies such as IoT-based solutions have been increasingly adopted. Despite the substantial initial costs, these wireless technologies offer significant long-term benefits, including enhanced operational efficiency, reduced maintenance costs, improved reliability, and increased transparency, aligning closely with sustainability and economic goals [3].

The authors in [2] proposed a transition from manual to automated operations at the water station to address the operational challenges. This system aims to minimize human error and enhance performance by monitoring tank water levels with float sensors, directing water flow as needed, and controlling pump activity based on pipeline pressure to prevent dry runs. The system utilizes a Programmable Logic Controller (PLC) at the water tanks to connect various field instruments, such as pressure switches, solenoid valves, differential pressure sensors, and float level switches. A key feature of the system is enabling the PLCs at each tank to communicate with each other to control the pumping and water flow between the two tanks, allowing them to feed each other.

To cover the wide area between the water tank and wells without extensive wiring and establish a reliable connection, the automation system uses a wireless network designed by [4]. This network places wireless modules at the wells to collect data and transmit it to the PLC using radio frequency (RF) signals, which in turn sends commands to control the water station’s operation.

However, as discussed by authors in [4], the wireless network designed separately from the automation system led to several operational issues. The biggest problem was that the secondary tank was completely neglected in the first wireless network design, which meant it was not integrated into the system. This omission resulted in significant gaps in monitoring and control for that tank, negatively impacting the overall efficiency of the water station. In addition, the equipment used in the initial wireless network design was not suited for IoT applications, and no prior testing or related work had been conducted using these devices in a similar environment. This raised concerns about the feasibility and reliability of the system, as the chosen equipment did not meet the necessary requirements for efficient operation in an IoT-based system.

Our research work meticulously reviews various wireless network designs and technologies, selecting (IEEE 802.11) [5] and Phoenix Contact’s Trusted Wireless as the most suitable technologies that meet the water system requirements within national operation regulations. It comprehensively examines the water station infrastructure and the distribution of the field instruments to propose two wireless networks for each technology. The general infrastructure consists of two water tanks, a main and a secondary, each supplied by surrounding wells at various distances. Therefore, the water station was split into two segments, one for each water tank.

The (IEEE 802.11) wireless network design in our work adopts a star topology for both segments, with instruments at the wells (remote nodes) communicating directly with the water tank (Base Station). In the first section, the star network is further divided into three sectors, each collecting data from a number of wells to allow scalability and prevent a single point of failure. The second section, which only has three wells to supply the secondary tank, utilizes one omnidirectional antenna to connect all the wells in that section.

The Trusted Wireless network enables the mesh topology and repeater functionality of its wireless modules. This allows the first section of the network to be designed as a cluster-tree topology, with certain wells acting as cluster heads that mediate between the remote nodes in their clusters and the base station (BS). Nodes can switch clusters and communicate with the base station through the CH with the best connection. The second section of the network adopts a star topology similar to the (IEEE 802.11) network.

Both designs were then validated using Radio Mobile, which performs link budget calculations between every two points of connection. It draws a terrain map to calculate the path loss based on the coordinates of the water station, accompanied by the parameters of the wireless modules, such as TX power, gain, and receiver sensitivity. These parameters were systematically adjusted until each link met a specific Received Signal Strength Indicator (RSSI).

This paper is organized as follows: Section 2 reviews related work in the area of network architectures and designs for deployment of industrial Internet of Things (IIoT) in real-life networks. Section 3 examines the water station infrastructure, followed by an exploration of possible topologies based on wireless technology features in Section 4. Section 5 proposes optimal network architectures and designs for the real-life water distribution network based on findings from the previous sections. Section 6 discusses mathematical models to validate the designs. Section 7 selects software Radio Mobile version 11.6.7 and wireless modules, with simulation results presented in Section 8. The discussion and conclusion are in Section 9 and Section 10.

## 2. Related Work

Automation systems used to rely on wired connections, but challenges like distance and environmental obstacles often make wireless networks a better option [6].

### 2.1. IEEE 802.15.4 Based

Zigbee, adhering to (IEEE 802.15.4) [7], is efficient in power use and suited for short-range communications, benefiting systems like those in agriculture developed by authors in [8]. Despite its advantages in creating energy-efficient, cost-effective networks, Zigbee’s limited range hinders its use in large-scale projects, such as water station automation.

For industrial applications, Ref. [9] used WirelessHART for temperature control and diagnostics by integrating it with PLCs and SCADA systems, enhancing real-time automation control. Although proven to be suitable for industry, it has the same range limitation as Zigbee and struggles with interoperability due to its exclusive support for HART devices, posing challenges for comprehensive system integration.

Reference [10] used ISA-100.11a in their design for water level management, through a system comprising sensors, a repeater, a wired actuator, and a controller, all coordinated via ISA-100.11a for operational stability. The system highlights ISA-100.11a’s capacity for maintaining network performance under industrial conditions, including real-time link stability adjustments. Despite its strengths, ISA-100.11a shares the range limitations of Zigbee and WirelessHART.

Addressing the range limitation, ref. [11] presented Yokogawa’s approach, enhancing range and reliability through a redundant wireless network with a mesh topology for broader coverage; the practicality of such an expansion is constrained by the network’s hop limit, potentially complicating the network and increasing costs, a significant limitation for the extensive range required by the Sof-Algeen water station.

### 2.2. IEEE 802.11-Based Solutions

Wi-Fi based on (IEEE 802.11) [5] plays a vital role in industrial settings, supporting operations with high data rates and long-distance connectivity through high-gain antennas. The authors of [6] demonstrated this in their design by integrating PLCs, Input/output (I/O) modules, and sensors for efficient water level management, indicating how Wi-Fi facilitates precise control and monitoring, supporting real-time data exchange.

### 2.3. Low-Power Wide-Area Network (LPWANs)

LoRaWAN stands out for its ability to support long-range communications and low power usage, crucial for developing efficient wide-area networks as demonstrated by [12], in their irrigation control system. However, the authors in [13] argue that because of LoRaWAN’s slow update rate, it is less suitable for real-time control.

Similarly, Sigfox is also known for its long-range communications and low-power consumption, as showcased by [14] in their smart trash bin system. Despite its benefits, Sigfox’s limited data transmission and vulnerability to interference may hinder its suitability for dynamic, real-time applications [15].

### 2.4. Trusted Wireless

Trusted Wireless, a wireless technology developed by Phoenix Contact, merges the real-time data handling of WirelessHART with the extensive reach of LoRaWAN and Sigfox. Individual studies on this technology have not been found. However, the manufacturer successfully provided several studies, where they successfully implemented it in an oil tank monitoring system covering a 20-mile desert radius, enabling automatic emergency shutdowns [16].

The regulatory insights provided by the Network Design departments of Libyana, a government-owned telecommunications company, and AL Jabal ISP, confirm that in Libya, only a limited number of channels in the 2.4 GHz range are accessible, while the full spectrum between 4 GHz and 6 GHz is available. This regulation excludes the use of Sigfox and LoRaWAN due to their reliance on low-frequency bands. Additionally, cellular-based communication technologies are restricted to government-owned companies, further limiting the feasibility of using these solutions for IoT applications.

Based on these findings, this project has selected IEEE 802.11-based (Wi-Fi) and Trusted Wireless technologies as viable options for designing the network.

## 3. Water Station Infrastructure

**Figure 1:**
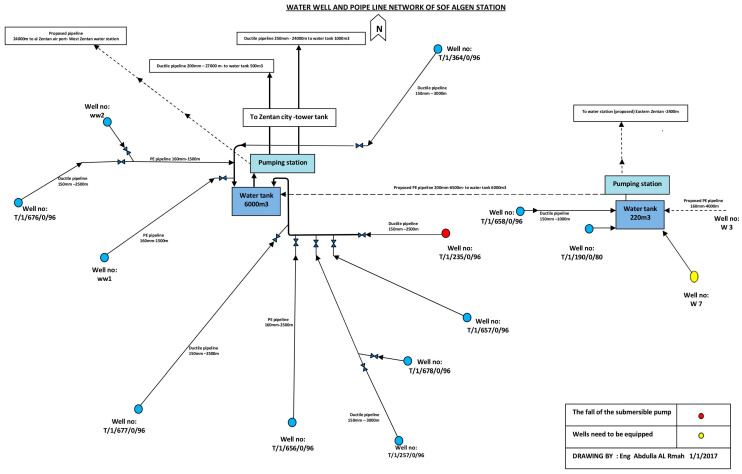
Water Well and Pipeline Network of Sof-Algeen Station—Archived Map from the Water and Sanitation Branch in Zintan City (Drawn by Eng. Abdulla AL Rmah, 1 January 2017).

The water station consists of two water tanks each surrounded by several wells at a distance. The main tank is supplied by ten wells, and the secondary by four wells, as shown in Figure 1. However, one of the wells supplying the secondary tank is merely a few meters away from the tank.

According to [2], the automation system design features two PLCs assigned to each tank, which are responsible for processing control signals to operate switches and valves throughout the network. This operation is guided by feedback from level sensors and differential pressure readings that monitor water levels within tanks and flow within the pipelines, a process made complex by the widespread distribution of the wells. Instruments located near the PLCs, such as the level sensors in the water tanks and the pressure switches of the centrifugal pumps, are directly wired, simplifying their integration.

Based on the automation system and the structure of the water station, determining the locations of the nodes in each well and using the water tanks as the base station (BS) where the PLCs are to be located, several network topologies can be proposed. These topologies are configurations for the wireless network, chosen depending on the wireless technology used (e.g., IEEE 802.11 or Trusted Wireless). They enable the wireless network to provide reliable and scalable communication, which in turn supports the automation system. The proposed network topologies are as follows:

Star topology: This configuration allows for easy setup and straightforward fault detection, as each device communicates through the central node. Its downfall is its reliance on the central node, as its failure can disable the entire network [17];

Mesh topology: in a mesh topology, nodes are not only connected to the central node but also to other nodes in the network, allowing data to take multiple paths from source to destination [17];

Cluster-tree topology: This topology combines elements of multiple star topologies into a larger, tree-like network [18]. In the water network, this topology could position the BS as the root, with clusters of wells as intermediary nodes extending to individual wells.

## 4. Wireless Technologies

After considering various network topologies, the focus now shifts to a thorough analysis of the selected wireless technologies. This examination prioritizes criteria such as network structure, transmission techniques, frequency bands, and security measures to validate their compatibility with the project’s requirements.

### 4.1. Wi-Fi

Wi-Fi is a wireless communication technology that allows devices to connect to a local area network. It is based on the (IEEE 802.11) [5], which specify how wireless communication is implemented in various frequency bands such as 2.4 GHz, 5 GHz, and recently 6 GHz for Wi-Fi 6. The (IEEE 802.11) has many variations, but the more recent ones are 802.11n (Wi-Fi 4), 802.11ac (Wi-Fi 5), and 802.11ax (Wi-Fi 6). The (IEEE 802.11) operates in the two lowest layers of the OSI (Open Systems Interconnection) model: The physical (PHY) layer is responsible for transmitting and receiving signals, as well as modulation and coding. The media access control (MAC) sublayer in the data link layer coordinates wireless medium access, security, and the structure of data packets [19,20].

(1) Network Structure: In (IEEE 802.11) [5], a group of devices communicating together are known as the basic service set (BSS). There are three main types of network deployment within these standards. For the infrastructure BSS, or just BSS, in this deployment, the devices are connected in the form of a star topology that connects all devices in the network to a BS known also as an access point AP. Additionally, there are also the ad-hoc BSS, which allows a peer-to-peer communication, and the Extended Service Set (ESS) to connect BSSs together through a backbone network [21].
(2) PHY and MAC Layer: For medium access, (IEEE 802.11) include two MAC mechanisms, also known as coordinating functions: The mandatory distributed coordination function (DCF), a contention-based MAC mechanism, is widely used and implemented in most commercial wireless modules. The optional point coordination function (PCF), polling-based and limited only to infrastructure-based networks (BSS), is used in applications that require low-latency and real-time communication [22].

Furthermore, the newer versions of the (IEEE 802.11) protocol are designed to work well with older versions in the same network. This is possible because the MAC sublayer basic functionalities remain interoperable across versions. Distinctions between versions are primarily introduced at the PHY layer, including modulation techniques, channel utilization, and transmission capabilities [20,23].

IEEE 802.11n, also known as (Wi-Fi 4), has a throughput reaching up to 600 Mbps. 802.11n uses an Orthogonal frequency division multiplexing (OFDM) modulation scheme for transmitting the data. It also introduced the multiple inputs multiple outputs (MIMO) technology by increasing the number of antennas at the sender and the receiver, resolving the multipath issue, and reducing bit error [23].

IEEE 802.11ac (Wi-Fi 5) builds upon the foundation of (Wi-Fi 4) improve the use of OFDM and MIMO and introduces the Multi-User MIMO, which allows an AP to communicate with multiple stations simultaneously rather than sequentially. These improvements enable 802.11ac to achieve data rates exceeding 1 Gbps [23].

IEEE 802.11ax (Wi-Fi 6) introduces Orthogonal Frequency Division Multiple Access (OFDMA), a key enhancement over the OFDM used in earlier standards. While OFDM efficiently transmits data from a single user across multiple subcarriers, OFDMA subdivides these subcarriers into smaller sub-channels. This allows the simultaneous allocation of bandwidth to multiple users, improving network efficiency, and achieving higher throughput [20,24].

(3) Security: Currently, the most used encryption protocols in Wi-Fi are Wi-Fi Protected Access 2 (WPA2) and Wi-Fi Protected Access 3 (WPA3). WPA2 utilizes AES encryption with Counter Mode Cipher Block Chaining Message Authentication Code Protocol (CCMP) for device authentication, and it uses Pre-Shared Keys (PSK) for the personal network. On the other hand, WPA3 improves upon WPA2 by implementing the Simultaneous Authentication of Equals (SAE) instead of the PSK [25].

### 4.2. Trusted Wireless Technology

Trusted Wireless technology developed by Phoenix Contact is a proprietary wireless alternative that bridges the gap between WirelessHART’s suitability for industrial applications and WLAN long-distance capabilities. It operates on several frequency bands: 868 MHz, 900 MHz, and 2.4 GHz. It can handle data rates of up to 250 Kbps, which is suitable for IIoT applications [26].

**Figure 2:**
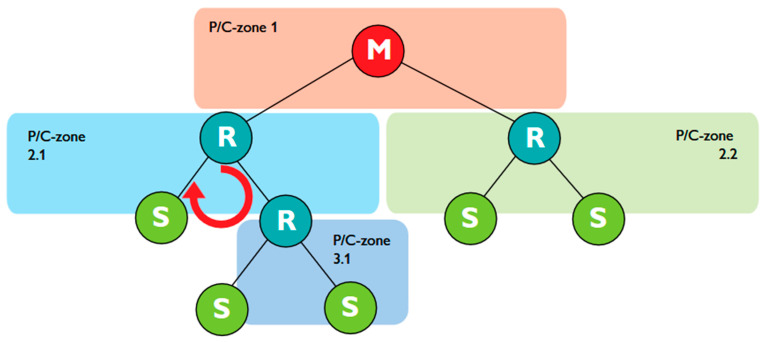
Distributed network in the parent–child zone [26].

(1) Network structure: Unlike Wi-Fi, Trusted Wireless supports mesh networking, allowing devices to serve as repeaters, thus enhancing communication range and reliability without direct paths to the base station (BS). Phoenix Contact, while not disclosing the routing protocol, introduced parent node black-listing and white-listing to optimize repeater roles and path planning to the BS. Furthermore, unlike WirelessHART, Trusted Wireless employs a more flexible, decentralized approach to network management using a parent–child relationship, as shown in Figure 2. Although the network design and data path are predefined, the parent–child zones mirror the Low-Energy Adaptive Clustering Hierarchy (LEACH) routing protocol’s cluster head approach [26].
(2) PHY and MAC Layer: For medium access, Trusted Wireless incorporates a Listen-Before-Talk (LBT) mechanism, which is the foundation of the Distributed Coordination Function (DCF) used in Wi-Fi, where devices sense the medium before transmitting to avoid collisions. Furthermore, like WirelessHART, Trusted Wireless employs FHSS as its transmission technology, hopping through up to 440 possible frequencies [26]. This makes the communication highly resilient to interference and less susceptible to jamming or interception, but compared to the OFDM and OFDMA used in Wi-Fi, it offers a lower data rate.
(3) Security: Trusted Wireless secures industrial communication in a similar manner to WirelessHART by employing AES-128 encryption in CCM mode and frequency hopping; it also utilizes a Pre-Shared Key (PSK) system for authentication, mirroring aspects of WPA2’s approach in Wi-Fi networks [26,27]. Compared to WPA2 and WirelessHART, Trusted Wireless use of frequency hopping and PSK combines the principles of robust encryption with practical key management, aiming for a balance between security and operational efficiency in industrial wireless networks.

## 5. Proposed Network Architecture Design

This section presents two wireless network architectures using (IEEE 802.11) and Trusted Wireless technologies for automating the Sof-Algeen water station. These technologies were selected due to their long-distance connectivity, reliable performance, and alignment with local regulatory standards.

Table 1 compares the selected technologies with existing alternatives, outlining the key differences in terms of area coverage, power consumption, data rate, and network topology, providing insight into how the proposed system compares with other technologies.

Based on the possible network topologies that can be implemented on the water station system, and the capabilities of the wireless technologies, two network architecture designs are proposed for Wi-Fi and Trusted Wireless to manage the operations of two separate water tanks and their associated wells: ten wells for the main water tank and four wells for the assistance tank, as shown in Table 2.

### 5.1. Network Architecture Using Wi-Fi Network Solution

For using Wi-Fi in industrial settings where data transmission reliability and timeliness are paramount, adopting a star topology through BSS is the preferred networking strategy. This configuration ensures superior Quality of Service (QoS), which is critical for operations where data from field instruments must be exchanged with the PLCs without delay.

**Figure 3:**
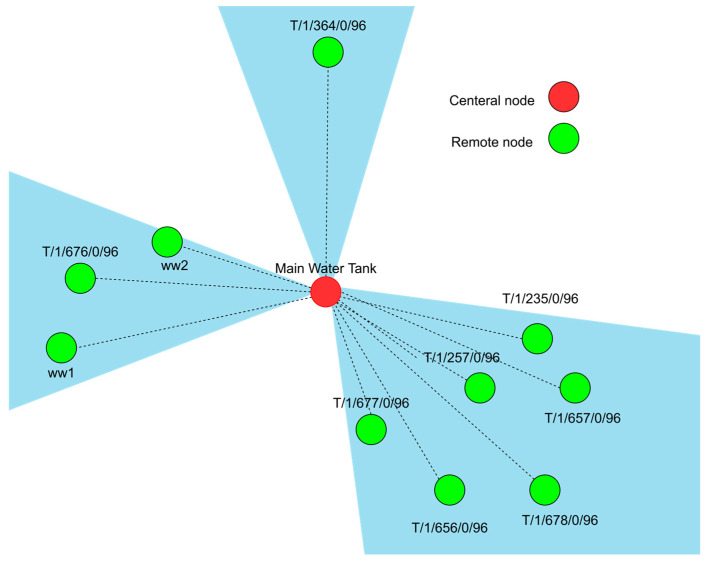
Illustration of the main water tank three-sector star network.

**Figure 4:**
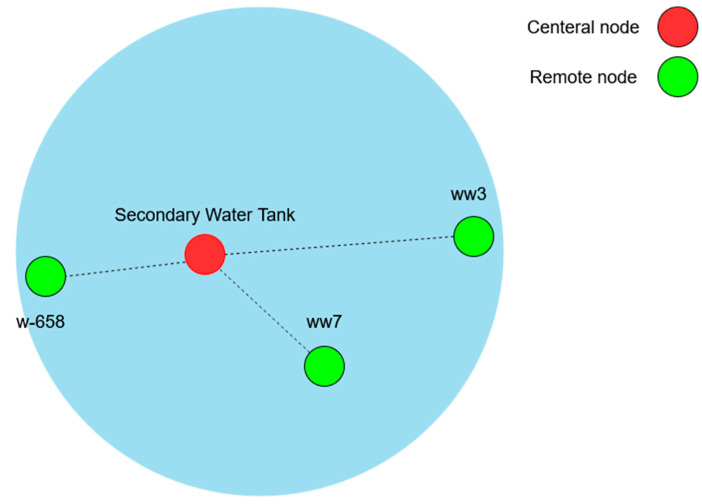
Illustration of the secondary water tank star network.

(1) Main Tank: Given that the PLC at the main tank monitors and controls data at ten wells, the network will be divided into three sections using sector antennas instead of a single omnidirectional antenna, as shown in Figure 3. Each cluster of wells will communicate with the PLC via its designated sector AP. This configuration not only helps avoid a single point of failure but also enhances network scalability.
(2) Secondary Tank: The secondary water tank adopts a star topology akin to the main tank but with a notable difference. It connects only three wells wirelessly because the fourth well (W-190) is directly wired due to its proximity. Given the smaller scale, the design proposes using an omnidirectional antenna as the AP, sufficient to cover the three wells as shown in Figure 4. Furthermore, to enable communication between the two PLCs at each network, a point-to-point link is required.

### 5.2. Network Architecture Using Trusted Wireless Network

Trusted Wireless technology supports the mesh topology, which allows distant nodes to communicate with the PLC by relaying data through intermediate nodes, shortening the distance for data transmission. The parent-listing feature of Trusted Wireless can help implement a hierarchical structure like that of the LEACH protocol, where nodes can serve as CHs, creating multiple paths for communication.

To maintain an efficient and reliable network topology, the Trusted Wireless network is designed with a three-hop limit, ensuring a balance between coverage, delay, and link reliability. This constraint prevents excessive latency and retransmissions, which can degrade network performance.

The challenge of optimizing multi-hop constraints has been widely explored in network topology design. Ref. [28] propose a constraint-based parallel local search algorithm to minimize path limits while preserving network robustness and connectivity. While this approach is highly effective for general large-scale networks, industrial automation networks require carefully controlled hop limits to maintain low latency and deterministic communication, as outlined by [11] in their implementation of ISA100 Wireless for industrial applications.

The three-hop constraint in our design follows a practical engineering trade-off, leveraging the long-range capabilities of Trusted Wireless to minimize hop counts while ensuring wide coverage. Even in future expansions, careful node placement will allow the system to maintain three-hop connectivity, preserving network efficiency without excessive reliance on relays.

(1) Main Tank: In this network, the PLC, serving as the central node, is directly connected to five repeaters. These repeaters function as CHs, coordinating communications with other remote nodes in the network and suggesting omnidirectional antennas to enable communication with the surrounding wells. However, in a few cases, like in the northern well (T/1/364/0/96), where there are no surrounding wells and it can only connect to the central mode, it is advisable to use a directional antenna.

**Figure 5:**
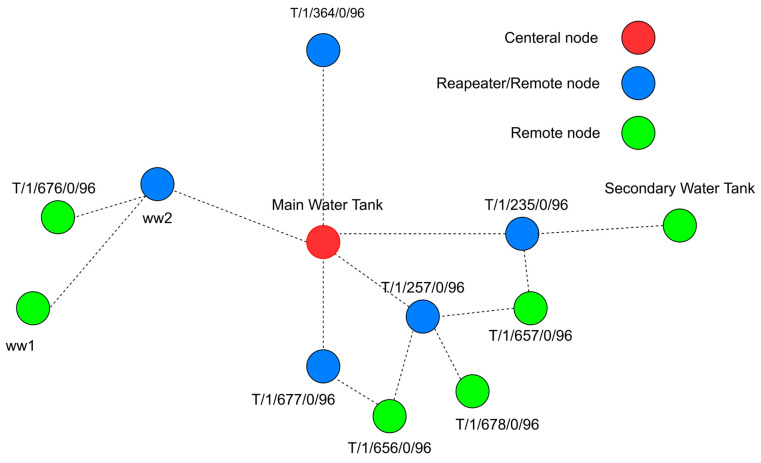
Illustration of the main water tank mesh network.

Additionally, Figure 5 highlights the integration of a node at the secondary tank into this network. This integration allows the PLC associated with the second tank to send control requests to the PLC in the main tank as necessary, in line with the control algorithm. This setup leverages the mesh networking capabilities serve as repeaters and clients. Hence, it eliminates the need for a direct point-to-point link between the two PLCs.

(2) Secondary Tank: For the second water station, due to the way the wells are distributed, a mesh topology will not be of any use. The best approach is similar to the one proposed in the Wi-Fi network design, which is to have a star that connects each well directly to the central node using directional high-gain antennas.

## 6. Mathematical Models

To assess the feasibility of previously proposed designs, especially for long distances, it is essential to perform a link budget calculation for every wireless link between two points. This calculation involves several key parameters of wireless modules, including transmit (TX) power, antenna gain, receiver sensitivity, operating frequency, and the distance between the two points. These factors collectively determine the ability of the communication link to maintain a reliable connection under specified conditions [29].

### 6.1. Free Space Loss

In addition to the previously mentioned parameter, free space loss (*FSL*) in wireless communication quantifies the attenuation of electromagnetic waves as they propagate through a vacuum. This attenuation is a critical factor in designing and analyzing wireless systems, directly influencing the received signal strength (*RSL*) over distance [29,30]. It can be calculated using the following formula:

(1) $$L_{FSPL}=-20{log}_{10}(\frac{4\pi D}{\lambda })$$

### 6.2. Receiver Signal Strength

**Figure 6:**
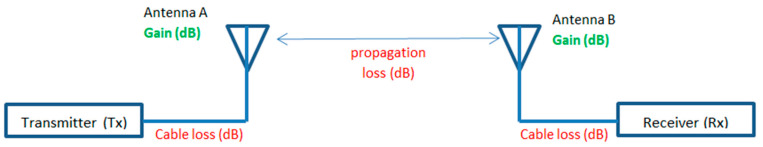
Wireless link budget components, showing transmitter power, antenna gain, and propagation loss [31].

With the path loss calculated, the link budget calculation, shown in Figure 6 is used to determine the *RSL*, which measures the arriving signal strength at the receiver in decibels relative to a milliwatt (dBm). *RSL* is essential for evaluating the quality and reliability of a communication link, ensuring the signal is strong enough for accurate interpretation by the receiver [29,30].

(2) $$RSL=P_{TX}+G_{Tx}-L_{Tx}+G_{Rx}-L_{Rx}{-L}_{FSPL}$$

### 6.3. Fresnel Zones

Path loss is crucial for assessing link feasibility and is easily calculated in free space, but environmental factors, like urban obstacles such as buildings, vehicles, and trees, can disrupt the path. Maintaining a Line-of-Sight (LoS) between the sender and receiver is vital as obstacles significantly increase path loss.

**Figure 7:**
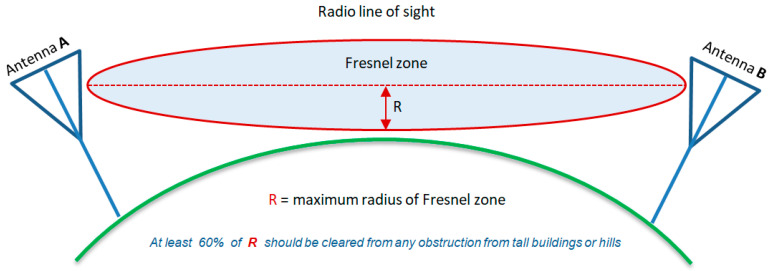
Fresnel zone representation, where at least 60% of the zone must remain unobstructed for optimal signal transmission [31].

Another critical factor is the Fresnel zones, elliptical areas around LoS between transmitter and receiver. As shown in Figure 7, Fresnel zones help identify where obstacles might cause signal phase issues. For optimal signal strength, at least 60% of the first Fresnel zone should be clear of obstacles, though more clearance is ideal to minimize interference and signal degradation [30,31]. It can be calculated as follows:

(3) $$Fn=\sqrt{\frac{n\lambda d_{1}d_{2}}{D}}$$

## 7. Software and Wireless Modules

### 7.1. Radio Mobile

Radio Mobile software version 11.6.7 is one of the most well-known tools in designing wireless networks. This tool is particularly noted for its precision in calculating link budgets for long-distance wireless networks, demonstrating its widespread adoption and effectiveness in determining link feasibility, as demonstrated by [32,33,34].

**Figure 8:**
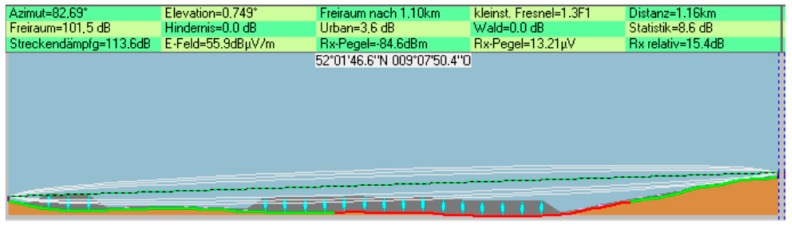
An image produced by Radio Mobile that shows the condition of a wireless link [32].

The software calculates path loss based on the terrain between the sender and receiver. Using the longitude and latitude of the location where the network is intended to be set, the software draws a digital image with natural obstacles, such as trees displaying the condition of the LoS and Fresnel zone, accompanied by the values of the link budget calculations. The tool’s ability to produce the image shown in Figure 8 hinges on the parameters of the wireless module at each location, such as TX power, antenna gain, and receiver sensitivity.

### 7.2. Wi-Fi Wireless Module

For the Wi-Fi network, Siemens SCALANCE WLANs are selected, as they are suitable and excel in the design of Wi-Fi wireless networks within industrial settings. As mentioned before, Wi-Fi uses PCF for medium access in applications focusing on QoS, by prioritizing real-time traffic. However, its practical effectiveness is limited. Therefore, Siemens provides a proprietary technology, known as Industrial Point Coordination Function (iPCF), to enhance QoS. iPCF proactively governs the network traffic by systematically polling each client according to a pre-determined sequence [35].

**Figure 9:**
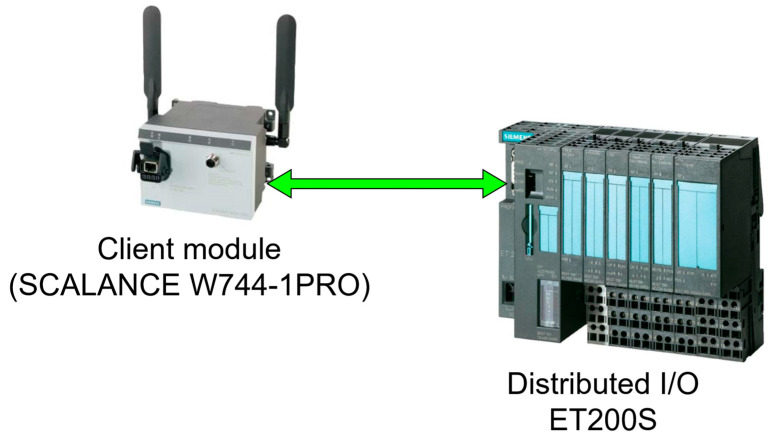
Siemens Wireless module connected to field devices via a Distributed I/O ET 200S [6].

Furthermore, to connect the wireless modules which are ethernet-based to the field instruments which in this case are I/O devices, a distribution I/O to act as a bridge between the two devices is needed, as shown in Figure 9, at each location, which will increase the cost significantly. However, some of the SCALANCE module such as SCALANCE WxM763, have Digital Input/Digital Output (DI/DO) ports that allow the connection of the devices directly.

### 7.3. Trusted Wireless Modules

**Figure 10:**
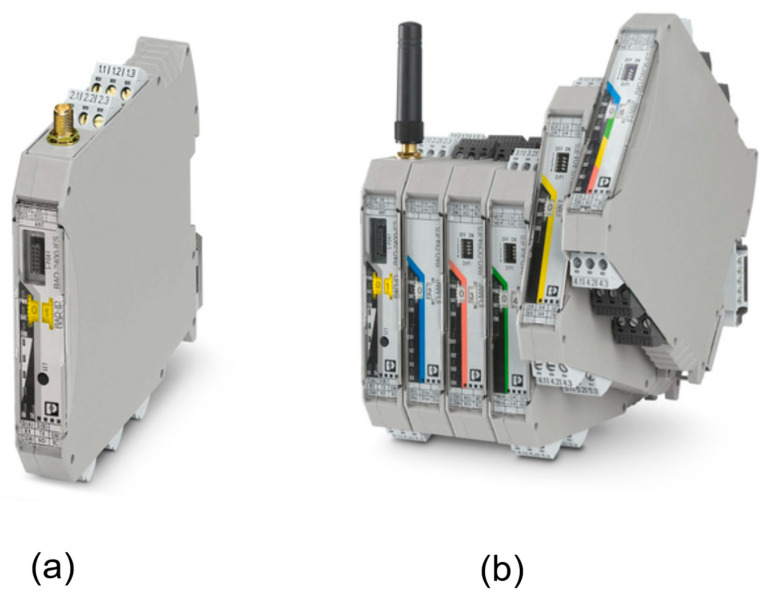
(**a**) RAD-2400-IFS wireless modules. (**b**) RAD-2400-IFS with an antenna installed and connected to several different extension I/O modules [36].

Trusted Wireless is a proprietary technology developed exclusively by Phoenix Contact. Therefore, the selection of wireless modules is inherently limited to Phoenix Contact’s Radioline module. There are three modules: RAD-868-IFS, RAD-900-IFS, and RAD-2400-IFS, operating at 800 MHz, 900 MHz, and 2400 MHz, respectively. Notably, regulatory restrictions in Libya narrow the selection to RAD-2400-IFS for 2.4 GHz. Furthermore, the RAD-2400-IFS wireless module, shown in Figure 10, has an RS-232/RS-485 interface and can be extended with additional I/O modules to enable connectivity with field instruments. However, the extension modules will come at an additional cost.

## 8. Simulation

The link budget simulation is crucial in network design, systematically evaluating performance under various conditions by adjusting transmission power and antenna height based on each antenna’s location relative to its AP. These adjustments affect path loss, influenced by terrain and distance. In a flat desert with minimal obstructions, all antennas are set to a height of 5 m, considering earth’s curvature and potential vehicle obstructions to maintain line-of-sight and signal propagation. This height aids Fresnel zone clearance, minimizing costs. The height increases only to counter first-zone obstructions, as taller structures are costlier. Otherwise, only TX power is adjusted to optimize RSSI.

### 8.1. Wi-Fi Wireless Network

Following the methodology outlined earlier, the connectivity between each Ap antenna and its associated stations will be systematically validated and tested. The goal of these adjustments is to enhance the RSSI sufficiently to achieve a minimum of a 10 dB margin above the receiver’s sensitivity. This process ensures that all links are robust and capable of maintaining stable communication across the network, which is vital for uninterrupted tank and water wells operation. Table 3 shows the link parameters for the Wi-Fi based on SCALANCE wireless modules.

#### 8.1.1. Main Tank Sector 1

**Figure 11:**
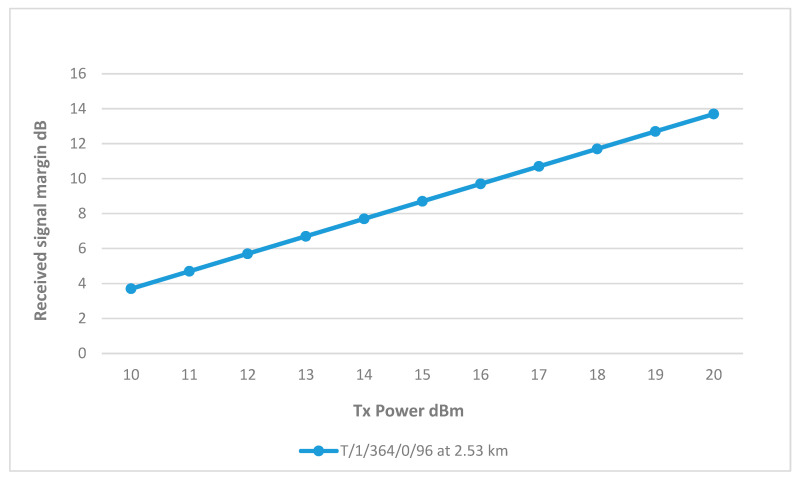
Transmit Power vs. RSSI for wells connected to Sector 1.

**Figure 12:**
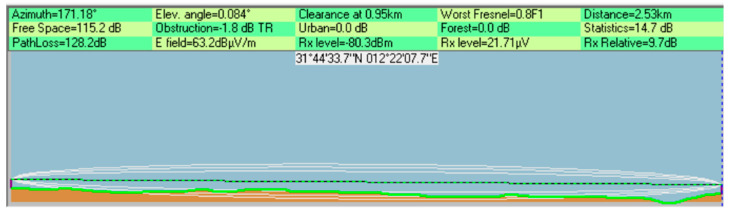
Radio Mobile result for well T/1/364/0/96 at 16 dBm.

Sector 1 is only connected to well T/1/364/0/96; as shown in Figure 11, the RSSI margin increases steadily with the Tx power and at 16 dBm, the RSSI was around 10 dB above the receiver sensitivity. Furthermore, Figure 12 shows a clear line of sight between the well and sector 1, with minimum obstruction in the Fresnel zone. Hence, the result is satisfactory, and the height will not be increased, as summarized in Table 4.

#### 8.1.2. Main Tank Sector 2

**Figure 13:**
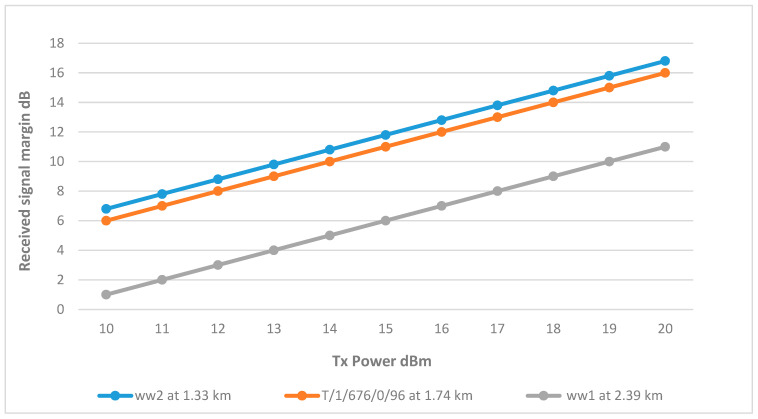
Transmit Power vs. RSSI for wells connected to Sector 2.

As shown in Figure 3, sector 2 covers three wells, which are ww1, ww2, and T/1/676/0/96. The results show that the links for wells ww2 and T/1/676/0/96 are achieving the minimum margin at 15 dBm. On the other hand, well ww1 requires a higher Tx power at 19 dBm, and the reason for this is that ww1 is further away from the BS at 2.39 km, resulting in a higher path loss due to the distance, difference in elevation, and terrain as shown in Figure 13.

**Figure 14:**
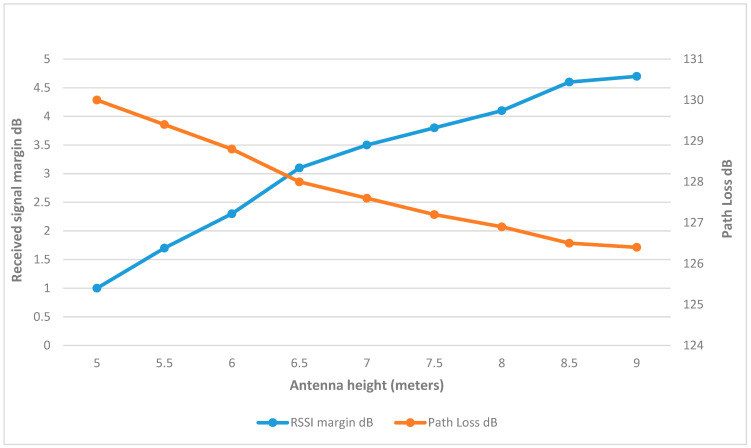
ww1 RSSI margin and path loss values at different heights.

**Figure 15:**
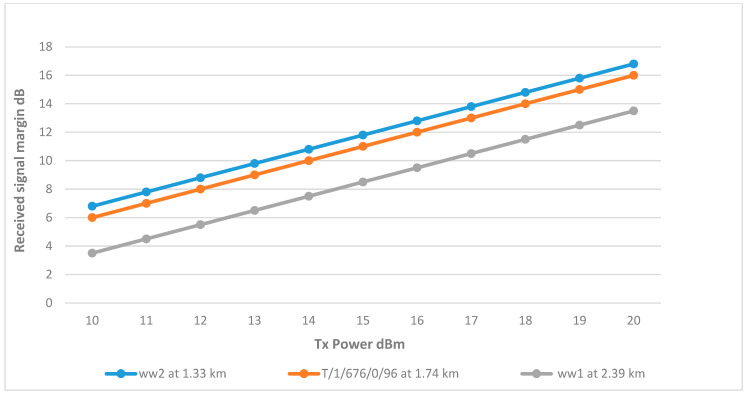
Transmit Power vs. RSSI for wells connected to Sector 2, with height adjustments.

Figure 14 demonstrates that the RSSI margin in ww1 increased by approximately 1 dB for each incremental rise of 0.5 m in antenna height, while the path loss decreased. This trend of increasing signal margin continued to a height of 7 m, beyond which the margin gains were only slight. Therefore, at this height, ww1 was able to reach the margin at 17 dBm, which is an improvement over the previous results as shown in Figure 15. The final link parameters are summarized in Table 5.

#### 8.1.3. Main Tank Sector 3

**Figure 16:**
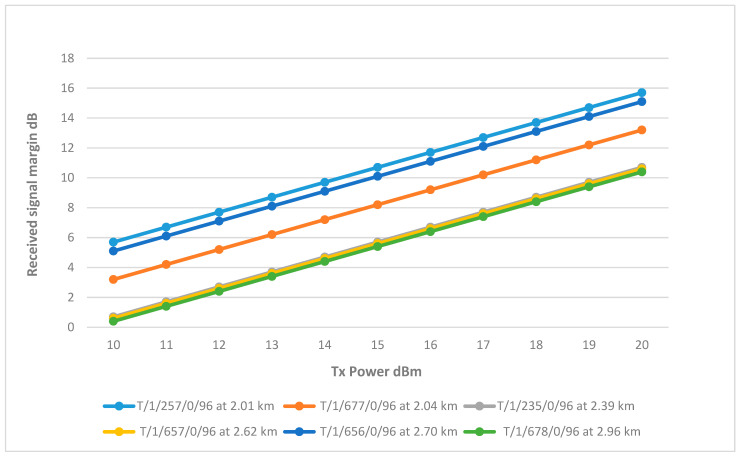
Transmit Power vs. RSSI for wells connected to Sector 3 with improved heights.

The results showed that wells T/1/257/0/96 and T/1/656/0/96 have relatively high RSSI at 15 dBm. T/1/677/0/96 slightly less, reaching the 10 dB threshold at 17 dBm. On the other hand, the RSSI of wells T/1/235/0/96, T/1/657/0/96, and T/1/678/0/96 is significantly lower. They only meet the threshold at full transmitter power. The differences in their performance can be attributed to their distance and terrain impact. Therefore, using the same approach implemented in sector 2, it was found that by increasing the height of the antennas, the RSSI improved as the path loss reduced. All three wells achieved good results at 7.5 m, reaching the RSSI margin at a TX power between 16–17 dBm, as shown in Figure 16. The final link parameters are summarized in Table 6.

#### 8.1.4. Secondary Tank Wi-Fi Network

**Figure 17:**
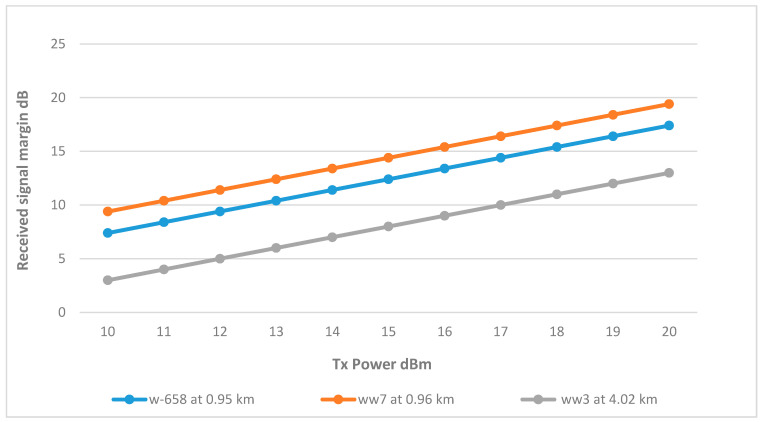
Transmit Power vs. RSSI for wells connected to the secondary tank at various distances.

Utilizing a single omnidirectional antenna to serve all three remote wells. Figure 17 shows wells w-658 and ww7 successfully achieved the RSSI margin at around 12 dBm. This can be attributed to their proximity to the tank, with distances just under 1 km. Conversely, well ww3, being the farthest at a distance of 4.02 km, necessitated a higher Tx power of 17 dBm to achieve an acceptable RSSI margin.

Given that the Fresnel zone in the ww3 link is free from obstructions, raising the antenna height did not improve the signal for well ww3. The alternative options to improve the link’s RSSI include installing a repeater antenna midway between the BS and ww3 or utilizing higher gain antennas to establish a dedicated point-to-point link. However, the ww3 link is considered viable at 17 dBm with the current antenna configuration. The final link parameters are summarized in Table 7.

#### 8.1.5. Connecting the Two Networks

The initial design proposes the establishment of a dedicated point-to-point link between the two tanks using high-gain directional antennas. However, the distance between the two tanks is considerable, nearly 7 km, causing the Earth’s curvature to significantly obstruct the Fresnel zone, compromising the integrity of the connection. To solve this issue, a taller structure is needed to have a clear Fresnel zone.

**Figure 18:**
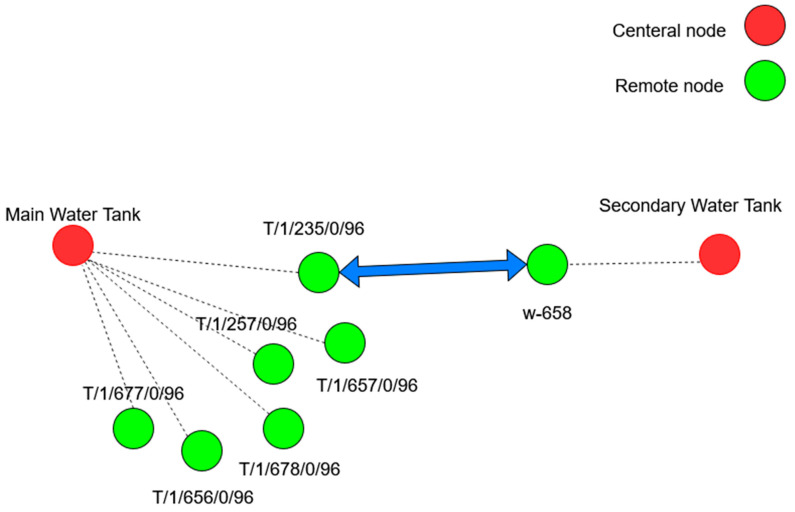
The link between well w-658 and well T/1/235/0/96.

However, considering the positioning of the wells shown in Figure 18, it is notable that well w-658, which is connected to the secondary tank, and well T/1/235/0/96, connected to the main tank, are aligned with each other. This alignment presents an opportunity for these wells to act as a bridge between the two networks.

By utilizing directional antennas with a Tx power of 10 dB and an antenna height of only 5 m, an RSSI margin of 10.9 dB is achieved, which exceeds the required threshold Table 8 shows the final link parameters. This approach eliminates the need for a taller structure and strategically uses the water network structure.

### 8.2. Trusted Wireless Network

Like Wi-Fi, Trusted Wireless simulation adjusts TX power and antenna height to meet network needs. Additionally, some wireless modules in Trusted Wireless function as both repeaters and remote nodes, requiring adjustments in TX power, antenna height, and type. Initially, all CHs and remote nodes will use the RAD-ISM-2400-ANT-OMNI-9 omnidirectional antenna with 9 dBi gain. Depending on performance outcomes, other antenna types may be considered. Table 9 shows the link parameters for the Trusted Wireless based on RAD-2400-IFS wireless modules.

In alignment with the proposed design for the main tank, the network will be segmented into CHs (repeaters) that communicate with the central node (BS) and subsequently forward data to and from remote nodes. The connection between BS and the CHs, which is the backbone of the network, needs to be resilient, given that losing one link will cut off a whole section of the network, unlike in the Wi-Fi star network where losing a link means that only one device was lost. Therefore, the RSSI margin will be increased to 15 dB instead of 10.

#### 8.2.1. Main Tank to Cluster Heads

**Figure 19:**
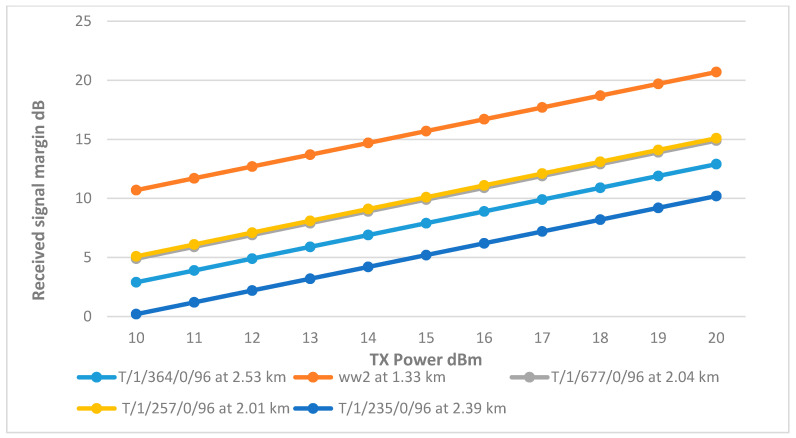
Transmit Power vs. RSSI plot for the main tank and the CHs.

This is the backbone of the entire network as it will connect the CHs to the BS at the main tank. Starting with an omnidirectional antenna of 9 dBi gain, Figure 19 shows that only CH ww2 was able to achieve the margin due to its proximity, while the rest of the CHs fail to meet this threshold even at full transmission power. Therefore, the other CHs will require antennas with higher gains to improve their signal strength.

Figure 5 shows that CH at T/1/364/0/96 isolated with no remote nodes to supervise would benefit from the directional RAD-ISM-2400-ANT-PAR-19-0 antenna, as it focuses the signal more effectively, enhancing connectivity. In contrast, other CHs managing data from remote nodes need the omnidirectional antenna with higher gain. Therefore, the Allendale Electronics omnidirectional antenna, offering a 15 dBi gain, will be used to improve connectivity.

**Figure 20:**
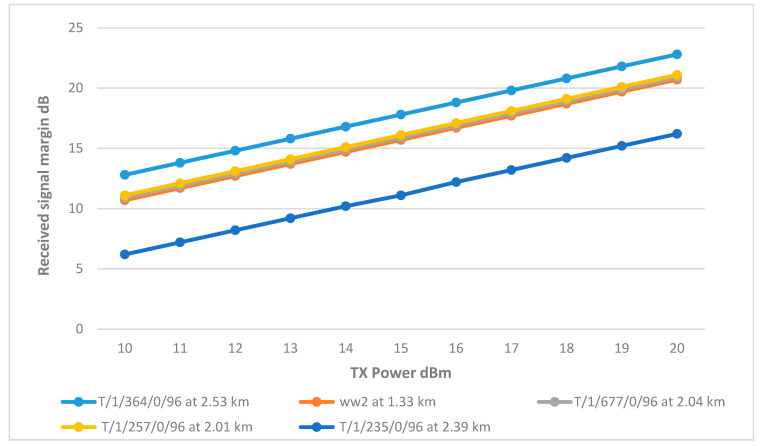
Transmit Power vs. RSSI plot for the main tank and the CHs with improved gain.

Figure 20 shows that the RSSI of the CHs has significantly improved with the use of higher gain antennas. CHs matched the performance of cluster head ww2, reaching the desired margin at a transmitter power of 15 dBm, with CH T/1/364/0/96 achieving the highest RSSI due to the use of a directional antenna.

**Figure 21:**
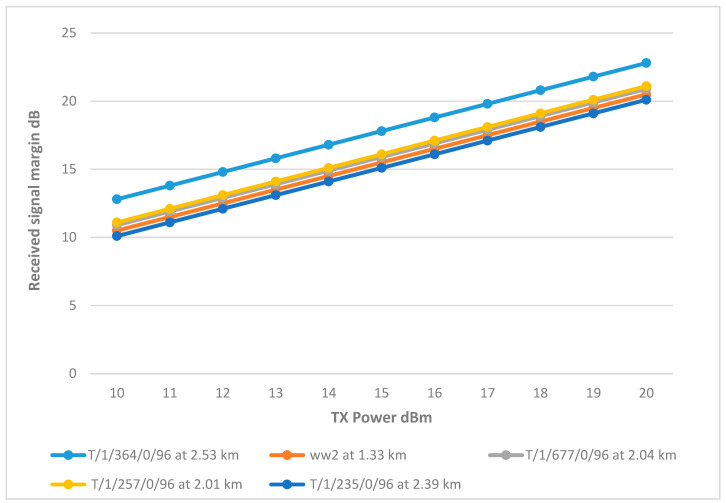
Transmit Power vs. RSSI plot for the main tank and the CHs with adjustment to gain and height.

However, CH T/1/235/0/96 displayed a significant drop in RSSI compared to the rest of the network, mirroring behavior observed in the Wi-Fi network. This drop is attributed to the antenna’s low height, which resulted in higher path loss due to terrain interference. To address this issue, increasing the antenna height to 8.5 m improved the RSSI, as documented in Figure 21. The final link parameters are summarized in Table 10.

#### 8.2.2. Cluster Head ww2

**Figure 22:**
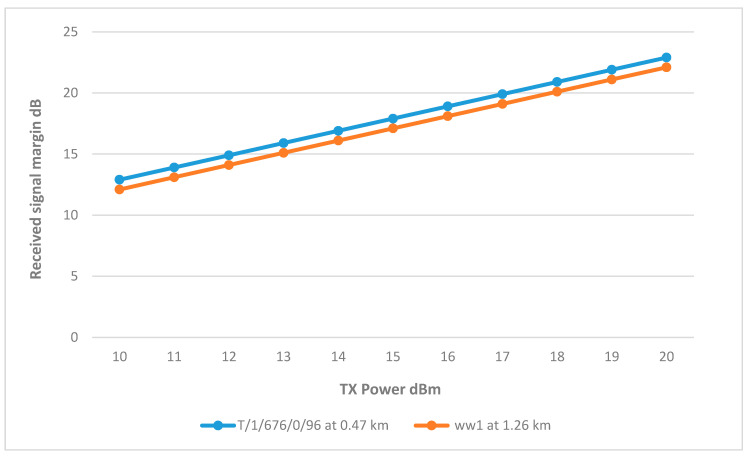
Transmit Power vs. RSSI plot for CH ww2.

ww2 is the only CH that is using a 9 dBi omni antenna, and is connected to remote nodes at well ww1. Well T/1/676/0/96 showed varying results, while T/1/676/0/96 achieved the margin at only 12 dBm due to its proximity to the CH. On the other hand, ww1 only reached the margin at full TX power, which is due to distance as no obstructions are found in the Fresnel Zone. Therefore, using the 15 dBi omni antenna, ww1 was able to achieve the margin at 13 dBm as shown in Figure 22. The final link parameters are summarized in Table 11.

#### 8.2.3. Cluster Head T/1/677/0/96

**Figure 23:**
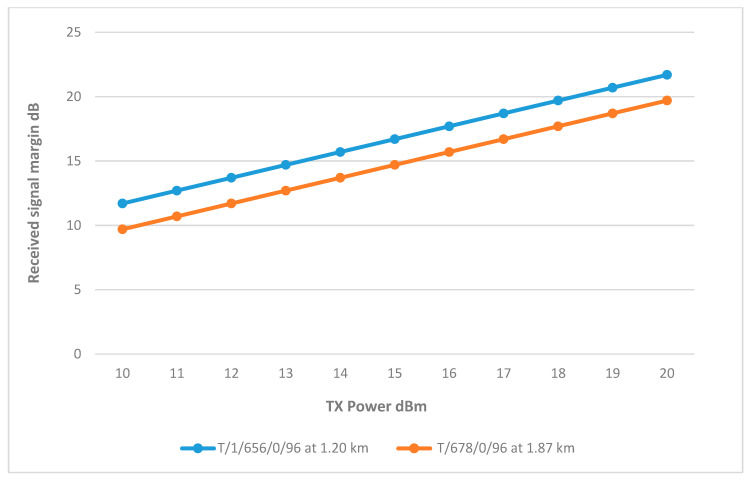
Transmit Power vs. RSSI plot for CH T/1/677/0/96.

Figure 23 shows that the connection between the CH and well T/1/656/0/96 is feasible, with the required margin being achieved at 14 dBm. Although not part of the initial design, the link between CH T/1/677/0/96 and well T/1/678/0/96 also performed well, reaching the margin at about 15 dBm using the same 9 dBi gain for both locations. This effective connectivity is partly attributed to the 15 dBi gain antenna used to connect the CH with the main tank, broadening its range to include remote nodes at a distance. The final link parameters are summarized in Table 12.

#### 8.2.4. Cluster Head T/1/257/0/96

**Figure 24:**
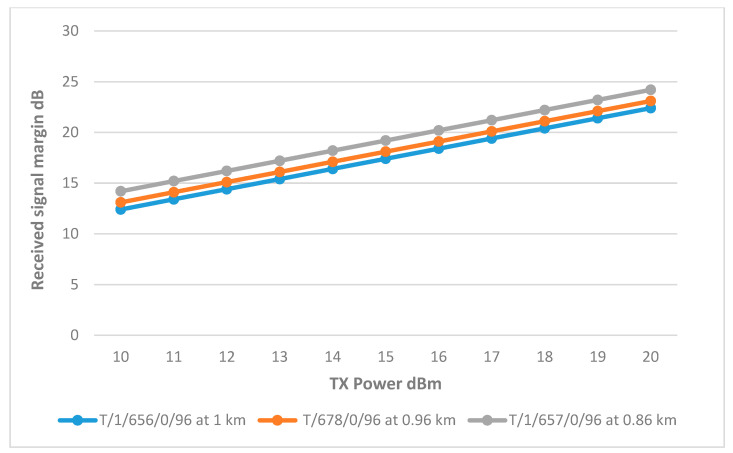
Transmit Power vs. RSSI plot for CH T/1/257/0/96.

Figure 24 illustrates that the remote nodes at wells T/1/657/0/96, T/1/678/0/96, and T/1/656/0/96 achieved the necessary margin at 11 dBm, 12 dBm, and 13 dBm, respectively. As shown in Table 13 the variation in transmitter power required to reach these margins is primarily due to the differences in distance between each remote node and the corresponding CH.

#### 8.2.5. Cluster Head T/1/235/0/96

CH T/1/235/0/96 plays a critical role in this network. According to the initial design, it connects to two remote nodes. The first, T/1/657/0/96, already has access to the central node through T/1/257/0/96. The second remote node is the secondary tank, which serves as a link between the two networks, considering the distance between the secondary tank and the CH, and as the secondary tank does not have any other route to connect to the main tank, the directional antenna was used.

**Figure 25:**
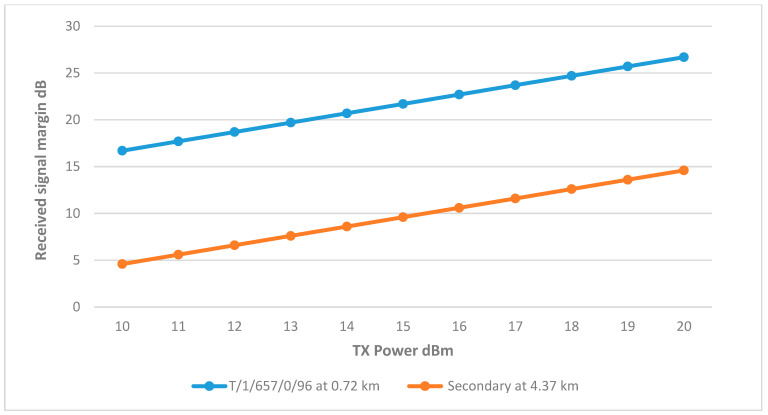
Transmit Power vs. RSSI plot for CH T/1/235/0/96.

The results in Figure 25 showed that the connection between the CH and well T/1/657/0/96 is above the margin at 10 dBm. However, the connection between the secondary tank and the cluster head was poor, with the margin not reached even with the directional antenna at full TX power, which is due to the earth’s curvature obstructing the Frensel zone, increasing the path loss and affecting the RSSI.

Since the antenna height at cluster head T/1/235/0/96 was previously increased to 8.5 m to connect it with the BS, it would be beneficial to increase the height of the directional antenna at the secondary to clear the Fresnel Zone from obstructions and reduce path loss.

**Figure 26:**
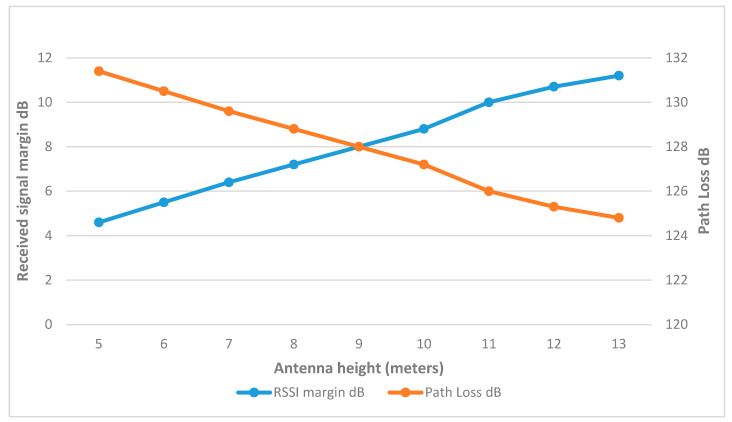
RSSI margin and path loss values at different heights.

Figure 26 shows that increasing the height up to 11 m made a significant improvement to RSSI. Testing this height at different TX powers showed promising results, as the margin was reached at 15 dBm. The final link parameters are summarized in Table 14.

#### 8.2.6. Secondary Tank Trusted Wireless Network

Since Trusted Wireless shares the same design as Wi-Fi for the secondary tank, and taking into consideration the Fresnel zone and distance challenges encountered previously in the Wi-Fi network simulation, for well ww3, which is 4.02 km from the BS, these challenges have been addressed by using a 15 dBi omnidirectional antenna at the center, and at ww3, the 19 dBi directional antenna has been employed to optimize connectivity. The other two wells, ww7 and w-658, are closer to the BS. Thus, the 9 dBi omnidirectional antenna is deemed sufficient.

**Figure 27:**
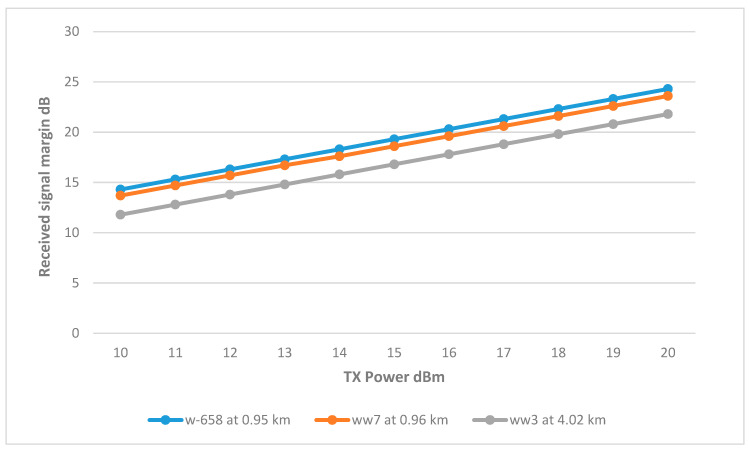
Transmit Power vs. RSSI plot for the secondary tank.

The results displayed in Figure 27 demonstrate that the selection of the antennas was strategic as all three remote nodes achieved the required margin at a relatively low transmission power of 12 dBm, with well ww3 requiring a slightly higher power of 13 dBm. The final link parameters are summarized in Table 15.

## 9. Discussion

### 9.1. Systematic Approach

The simulation systematically explored network performance by analyzing parameter variations. A 10 dB RSSI margin was set for the Wi-Fi network, following industry best practices for ensuring stable wireless communication. For Trusted Wireless, a 15 dB margin was chosen to account for multi-hop signal degradation, enhancing network reliability in industrial settings. An 80% Fresnel Zone clearance was maintained, aligning with standard recommendations to minimize diffraction losses and maintain strong signal integrity over long distances. These thresholds ensure resilience against environmental factors such as interference and extreme weather [32,33,34].

This method enabled the implementation of proposed designs using the minimal transmission power and antenna gain required to minimize unwanted noise. However, in certain cases, such as sectors 2 and 3 of the Wi-Fi network and cluster head T/1/235/0/96 in the Trusted Wireless network, the use of taller structures was unavoidable and necessary due to challenges like long distances, elevation differences, and terrain that obstructed the Fresnel zone.

### 9.2. Infrastructure

The water station infrastructure significantly impacted network performance. In the Wi-Fi network, the distribution of the wells was advantageous, enabling the division of the main tank network into three sectors. This division allowed for the use of higher gain, more directional sector antennas rather than standard omnidirectional ones, also preventing a total network failure if one sector malfunctioned, as the others could continue operating.

Conversely, for the Trusted Wireless Cluster Tree network, the infrastructure posed challenges to this topology. As seen in the proposed design, CHs T/1/677/0/96, T/1/257/0/96, and T/1/235/0/96 are closely positioned, and their remote nodes have multiple paths to the Base Station (BS). However, CH ww2 acts as a bottleneck, as its remote nodes lack alternative routes to the BS, meaning a loss of link to this CH could result in the loss of an entire section.

Additionally, the arrangement of the wells in relation to the secondary tanks precludes the use of a mesh approach, restricting the network to a star topology, which limits the functionality of Trusted Wireless. Despite these limitations, Trusted Wireless’s mesh capability proved advantageous in linking the two networks, unlike Wi-Fi, which required a dedicated point-to-point link.

### 9.3. Performance Comparison

Given that both wireless networks utilize the same design of the secondary tank, this offers a comparative basis to evaluate the following aspects:

#### 9.3.1. Frequency

In terms of performance, Wi-Fi operates within the 2.4 GHz and 5 GHz frequency bands, both of which comply with national telecommunications standards. The 2.4 GHz band typically offers fewer free channels compared to the 5 GHz band. Designing a network to operate at 5 GHz generally results in better connectivity due to the availability of more channels, enhancing the network’s flexibility to switch channels in order to mitigate noise and interference issues. Conversely, Trusted Wireless technology primarily operates on the 2.4 GHz band, with the lower frequencies (800, 900 MHz) being restricted. Although Trusted Wireless may exhibit better connectivity at lower frequencies, which can penetrate obstacles more effectively, it is also more susceptible to noise compared to higher frequency bands.

#### 9.3.2. Antenna Options and Connectivity

When discussing external antennas, Siemens does not specify the compatibility of its SCALANCE wireless modules with various external antennas and offers only a limited selection. The gains of their omnidirectional and sector antennas are relatively low, at 8 and 9 dBi, respectively. This necessitated the use of high-gain directional antennas, boasting 23 dBi, especially in the locations of the wells. Considering the use of different types of antennas, the link budget calculations were performed in both directions, from the base station to the wells and vice versa.

In contrast, Phoenix Contact’s Trusted Wireless technology explicitly supports the use of different external antennas. This flexibility enables the integration of high-gain omnidirectional antennas, significantly enhancing link connectivity. Such a feature is crucial, particularly as CHs and remote nodes must transmit signals in various directions to facilitate data transmission and explore alternate paths to the BS.

#### 9.3.3. Receiver Sensitivity and Data Rate

The RAD-2400-IFS offers a receiver sensitivity of −93 dBm at a data rate of 250 kbps, marking the lowest possible sensitivity at the highest data rate achievable by the Radioline series. This indicates that the Radioline could potentially reach higher sensitivity at reduced data rates. Nonetheless, the 250-kbps rate adequately meets the current application’s needs. In comparison, the SCALANCE wireless modules exhibit a receiver sensitivity of −90 dBm, which is the highest sensitivity comparable to the lowest that Trusted Wireless can offer. At this level of sensitivity, SCALANCE supports a significantly higher transmission rate of 15 Mbps, far surpassing that provided by Trusted Wireless. Although SCALANCE is capable of higher data rates, achieving these would necessitate a reduction in sensitivity, which may prove challenging given the extensive coverage area of the water station.

#### 9.3.4. Economic Considerations and Practical Implications

Both wireless networks explored in this study demonstrate distinct advantages depending on the specific operational context of the water station. To facilitate a clear understanding of these differences, Table 16 qualitatively summarizes key practical factors such as initial investment, scalability, complexity of installation, maintenance requirements, and long-term reliability for the (IEEE 802.11) and Trusted Wireless configurations. This comparison highlights essential considerations for effectively selecting the most suitable technology for robust, long-range water distribution network automation.

### 9.4. Deployment Challenges

The deployment of the proposed wireless network must consider several practical challenges. One of the key factors is ensuring a stable power supply for remote modules. As demonstrated in the proposed design, all active well sites will be connected to the main power line, ensuring continuous operation. However, to mitigate potential blackouts, backup battery systems are recommended to maintain network functionality during outages.

Another consideration is network interference. Given that the water station is located 30 km south of the city of Zintan [2] in an uninhabited area, the presence of external wireless networks is unlikely, reducing the risk of interference from neighboring networks.

Finally, the long-term maintenance of the network will depend on operational conditions and the collaboration between the water station management and the automation system design team. Regular maintenance activities may include periodic antenna realignment, firmware updates, and hardware replacements. These efforts will be crucial to sustaining optimal network performance over time.

## 10. Conclusions

This piece of research effectively addresses the limitations and gaps identified in previous wireless network design for a real-life water distribution network. It justifies the selection of specific wireless technologies, bridging the connectivity gap between wireless modules and field devices to ensure compatibility and system integrity. The integration of the secondary water tank into the wireless network was successfully executed. Moreover, the designs were systematically and rigorously validated, exploring various approaches, and maintaining a defined threshold of functionality across two distinct networks with different designs and behaviors.

Trusted Wireless, though less commonly used, showcased its strengths through a flexible design enabled by its mesh topology and parent–child communication strategy. The capability to incorporate various external antennas is particularly advantageous, allowing for tailored configurations that enhance network coverage and signal quality. In contrast, Wi-Fi technology excelled in providing higher data rates and operating across a wider range of frequency bands. This versatility is instrumental in reducing interference, a common challenge in densely populated network environments.

A notable limitation of this research lies in the performance testing of the two networks concerning error rates and noise levels. Due to the proprietary nature of SCALANCE’s iPCF medium access control mechanism and the lack of simulation support for Trusted Wireless, comprehensive performance assessments could not be conducted using simulation software. Future studies should focus on empirical testing under controlled conditions to validate the theoretical models and simulations presented in this work.

For future developments, high-throughput Wi-Fi could be utilized to enhance security at the water station, particularly through the deployment of advanced security cameras. Additionally, integrating the automation network with a cloud computing provider such as Amazon Web Services (AWS) could significantly improve monitoring and control capabilities. However, it is crucial to rigorously assess and mitigate potential security vulnerabilities associated with this enhancement.

Furthermore, future research could explore hybrid solutions that combine the strengths of both wireless technologies. By integrating Trusted Wireless for its robust mesh topology and long-range capabilities with Wi-Fi’s high data rates and broader frequency options, a hybrid approach could improve overall network performance, reliability, and scalability in water distribution automation.

## Figures and Tables

**Table 1:** Comparison of Wireless Technologies.

Technology	Area Coverage	Power Consumption	Data Rate	Network Topology	Strengths	Limitations
IEEE 802.11 (Wi-Fi)	30–100 m indoors, and up to 92 m outdoors	High: high throughput, drains battery quickly	Up to 54 Mbps	Star (access point)	High data rates, widely used, and easy integration	High power usage, limited range, and interference at 2.4 GHz
Trusted Wireless	Up to 1 km at 2.4 GHz, and up to 32 km at 900 MHz	Medium: efficient, but higher than Zigbee	Up to 250 kbps	Flexible (point-to-point, star)	Long distance, secure, and versatile topologies	Moderate data rate and higher power consumption than Zigbee
Zigbee	10–100 m per hop	Low: very low-power, long battery life	Up to 250 kbps	Mesh (self-organizing)	Low power and good for sensor networks	Short range and low throughput
WirelessHART	50 m typical, up to 250 m line-of-sight	Low: low power, battery-operated	250 kbps	Mesh (multi-hop)	Reliable for industrial monitoring, and deterministic timing	Short range and low data rates
LoRaWAN	5 km in urban, and 15+ km in rural areas	Very low: long battery life, and low duty cycle	Up to 50 kbps	Star-of-stars	Very long range, low power, and excellent penetration	Low data rate and high latency
Sigfox	10 km in urban, and up to 40 km in rural areas	Very low: can last 5–10 years on a battery	Up to 0.1 kbps	Star	Longest range, low power usage, and cost-effective	Very low throughput and no real-time support

**Table 2:** Water tanks associated wells.

Main Water Tank		Secondary Water Tank	
1.	T/1/677/0/96	1.	w-658
2.	T/1/656/0/96	2.	ww3
3.	T/1/257/0/96	3.	ww7
4.	T/1/678/0/96	4.	w-190
5.	T/1/657/0/96		
6.	T/1/235/0/96		
7.	ww1		
8.	T/1/676/0/96		
9.	ww2		

**Table 3:** Wi-Fi wireless network link parameters.

**Main and Secondary Tanks**	
Frequency	5000–5900 MHz
Antenna height	5 m
Wireless module (AP)	SCALANCE WAM763-1 (Wi-Fi 6)/W788-1 RJ45 (Wi-Fi 4)
Antenna	Sector antenna, ANT793-6DG, 9 dBi; omnidirectional antenna, ANT795-6M, 8 dBi
Transmission power	20 dBm
Receiver sensitivity	90 dBm
**Wells Locations**	
Frequency	5000–5900 MHz
Antenna height	5 m (vary)
Wireless module (clients)	SCALANCE WUM763-1 (Wi-Fi 6)/W748-1 RJ45 (Wi-Fi 4)
Antenna	Directional antenna: ANT793-8DK, 23 dBi
Transmission power	1–20 dBm (vary)
Receiver sensitivity	90 dBm

**Table 4:** Link Parameters for Sector 1.

Sector/Link	Distance (km)	Antenna Type (Base)	Antenna Type (Remote Node)	TX Power (dBm)	Antenna Height (m)	RSSI Margin (dB)	Fresnel Clearance (%)	Status
Main Tank Sector 1		ANT793-6DG			5			
T/1/364/0/96	2.53	ANT793-6DG	ANT793-8DK	16	5	10	~80	Feasible

**Table 5:** Link Parameters for Sector 2.

Sector/Link	Distance (km)	Antenna Type (Base)	Antenna Type (Remote Node)	TX Power (dBm)	Antenna Height (m)	RSSI Margin (dB)	Fresnel Clearance (%)	Status
Main Tank Sector 2		ANT793-6DG			5			
ww1	2.39	ANT793-6DG	ANT793-8DK	17	7	10.5	~80	Height adjusted, feasible
ww2	1.33	ANT793-6DG	ANT793-8DK	15	5	11.8	~80	Feasible
T/1/676/0/96	1.74	ANT793-6DG	ANT793-8DK	15	5	11	~80	Feasible

**Table 6:** Link Parameters for Sector 3.

Sector/Link	Distance (km)	Antenna Type (Base)	Antenna Type (Remote Node)	TX Power (dBm)	Antenna Height (m)	RSSI Margin (dB)	Fresnel Clearance (%)	Status
Main Tank Sector 3		ANT793-6DG			5			
T/1/257/0/96	2.01	ANT793-6DG	ANT793-8DK	15	5	10.7	~80	Feasible
T/1/656/0/96	2.70	ANT793-6DG	ANT793-8DK	15	5	10.1	~80	Feasible
T/1/677/0/96	2.04	ANT793-6DG	ANT793-8DK	17	5	10.2	~80	Feasible
T/1/235/0/96	2.39	ANT793-6DG	ANT793-8DK	16	7.5	10.1	~80	Height adjusted, feasible
T/1/657/0/96	2.62	ANT793-6DG	ANT793-8DK	17	7.5	10.1	~80	Height adjusted, feasible
T/1/678/0/96	2.96	ANT793-6DG	ANT793-8DK	17	7.5	10.4	~80	Height adjusted, feasible

**Table 7:** Wi-Fi network Link Parameters for Secondary Tank.

Sector/Link	Distance (km)	Antenna Type (Base)	Antenna Type (Remote Node)	TX Power (dBm)	Antenna Height (m)	RSSI Margin (dB)	Fresnel Clearance (%)	Status
Secondary Tank		ANT795-6M			5			
w-658	0.89	ANT795-6M	ANT793-8DK	13	5	10.4	~80	Feasible
ww7	0.96	ANT795-6M	ANT793-8DK	11	5	10.4	~80	Feasible
ww3	4.02	ANT795-6M	ANT793-8DK	17	5	10	~80	Feasible

**Table 8:** Link Parameters for the Relay Link Between Tanks.

Sector/Link	Distance (km)	Antenna Type (Base)	Antenna Type (Remote Node)	TX Power (dBm)	Antenna Height (m)	RSSI Margin (dB)	Fresnel Clearance (%)	Status
w-658 to T/1/235/0/96	3.43	ANT793-8DK	ANT793-8DK	10	5	10.9	~80	Feasible (relay)

**Table 9:** Trusted Wireless network link parameters.

Base Station, Remote Node, and Repeater	
Frequency	2000–2400 MHz
Antenna height	5 m (vary)
Wireless module	RAD-2400-IFS
Antennas	Allendale Electronics’ Omnidirectional (15 dBi), RAD-ISM-2400-ANT-OMNI-9 (9 dBi), RAD-ISM-2400-ANT-PAR-19 (19 dBi)
Transmission power	1–20 dBm (vary)
Receiver sensitivity	−93 dBm (250 Kbps)

**Table 10:** Link Parameters for Main Tank to Cluster Heads.

Sector/Link	Distance (km)	Antenna Type	TX Power (dBm)	Antenna Height (m)	RSSI Margin (dB)	Fresnel Clearance (%)	Status
Main Tank to CHs		RAD-ISM-2400-ANT-OMNI-9		5			
ww2	1.33	RAD-ISM-2400-ANT-OMNI-9	15	5	15.5	~80	Feasible
T/1/364/0/96	2.53	RAD-ISM-2400-ANT-PAR-19 (Directional)	13	5	15.8	~80	Feasible
T/1/677/0/96	2.04	Allendale Electronics’ Omnidirectional	15	5	15.9	~80	Feasible
T/1/257/0/96	2.01	Allendale Electronics’ Omnidirectional	15	5	16.1	~80	Feasible
T/1/235/0/96	2.39	Allendale Electronics’ Omnidirectional	15	8.5	15.1	~80	Height adjusted, feasible

**Table 11:** Link Parameters for Cluster Head ww2.

Sector/Link	Distance (km)	Antenna Type	TX Power (dBm)	Antenna Height (m)	RSSI Margin (dB)	Fresnel Clearance (%)	Status
Cluster Head ww2		RAD-ISM-2400-ANT-OMNI-9		5			
ww1	1.26	Allendale Electronics’ Omnidirectional	13	5	15.1	~80	Feasible
T/1/676/0/96	0.47	RAD-ISM-2400-ANT-OMNI-9	12	5	~15 (14.9)	~80	Feasible

**Table 12:** Link Parameters for Cluster Head T/1/677/0/96.

Sector/Link	Distance (km)	Antenna Type	TX Power (dBm)	Antenna Height (m)	RSSI Margin (dB)	Fresnel Clearance (%)	Status
Cluster Head T/1/677/0/96		Allendale Electronics’ Omnidirectional		5			
T/1/656/0/96	1.20	RAD-ISM-2400-ANT-OMNI-9	14	5	15.7	~80	Feasible
T/1/678/0/96	1.87	RAD-ISM-2400-ANT-OMNI-9	15	5	~15 (14.7)	~80	Feasible

**Table 13:** Link Parameters for Cluster Head T/1/257/0/96.

Sector/Link	Distance (km)	Antenna Type	TX Power (dBm)	Antenna Height (m)	RSSI Margin (dB)	Fresnel Clearance (%)	Status
Cluster Head T/1/257/0/96		Allendale Electronics’ Omnidirectional		5			
T/1/657/0/96	0.86	RAD-ISM-2400-ANT-OMNI-9	11	5	15.2	~80	Feasible
T/1/678/0/96	0.96	RAD-ISM-2400-ANT-OMNI-9	12	5	15.1	~80	Feasible
T/1/656/0/96	1	RAD-ISM-2400-ANT-OMNI-9	13	5	15.4	~80	Feasible

**Table 14:** Link Parameters for Cluster Head T/1/235/0/96.

Sector/Link	Distance (km)	Antenna Type	TX Power (dBm)	Antenna Height (m)	RSSI Margin (dB)	Fresnel Clearance (%)	Status
Cluster Head T/1/235/0/96		Allendale Electronics’ Omnidirectional		8.5			
T/1/657/0/96	0.72	RAD-ISM-2400-ANT-OMNI-9	10	5	16.7	~80	Feasible
Secondary Tank	4.37	RAD-ISM-2400-ANT-PAR-19 (Directional)	15	11	15	~80	Feasible

**Table 15:** Trusted Wireless network Link Parameters for Secondary Tank.

Sector/Link	Distance (km)	Antenna Type	TX Power (dBm)	Antenna Height (m)	RSSI Margin (dB)	Fresnel Clearance (%)	Status
Secondary Tank		Allendale Electronics’ Omnidirectional					
w-658	0.95	RAD-ISM-2400-ANT-OMNI-9	11	5	15.3	~80	Feasible
ww7	0.96	RAD-ISM-2400-ANT-OMNI-9	12	5	15.7	~80	Feasible
ww3	4.02	RAD-ISM-2400-ANT-PAR-19 (Directional)	14	5	15.8	~80	Feasible

**Table 16:** Comparative Analysis of Wireless Network Solutions.

Cost Factor	Wi-Fi (IEEE 802.11)	Trusted Wireless (Mesh)
Initial investment	Moderate	Higher
Installation complexity	Simple to moderate	Moderate to complex (mesh structure)
Network scalability	Good	Excellent (mesh support)
Maintenance complexity	Moderate	Lower (self-healing)
Long-term reliability	Good	High
Cost	Lower (widely available components and no licensing fees)	Higher (specialized hardware and potential licensing costs)

## Data Availability

The raw data supporting the conclusions of this article will be made available by the authors on request.

## References

[1] Imran M.A., Hussain S., Abbas Q.H. (2019). Wireless Automation as an Enabler for the Next Industrial Revolution.

[2] Alaribi A., Elazhari A., Zargelin O.A. PLC Based a Robust Solution for an Urban Area Water System Dilemma. Proceedings of the 2021 IEEE 11th Annual Computing and Communication Workshop and Conference (CCWC).

[3] Hristova T., Stoyanov I., Evstatiev B. (2023). Increasing the Functionality of Water Supply Systems for Bulgarian Conditions through Automation via IoT and Blockchain Technologies. Eng. Proc..

[4] Nagasa M.M., Alrmah M.A., Mussa M.A., Zargelin O.A. (2019). Designing Wireless Network for Water Issue in the City of Zintan. Int. Sci. Technol. J..

[5] (2020). IEEE Standard for Information Technology—Telecommunications and Information Exchange Between Systems—Local and Metropolitan Area Networks—Specific Requirements—Part 11: Wireless LAN Medium Access Control (MAC) and Physical Layer (PHY) Specifications.

[6] Bayindir R., Cetinceviz Y. (2011). A water pumping control system with a programmable logic controller (PLC) and industrial wireless modules for industrial plants—An experimental setup. ISA Trans..

[7] Koodtalang W., Sangsuwan T. Agricultural Monitoring System with Zigbee Network and PLC based on Modbus RTU Protocol. Proceedings of the 2020 International Conference on Power, Energy and Innovations (ICPEI).

[8] Thepmanee T., Pongswatd S., Asadi F., Ukakimaparn P. (2022). Implementation of control and SCADA system: Case study of Allen Bradley PLC by using Wireless HART to temperature control and device diagnostic. Energy Rep..

[9] Heitor F., Neto A.D., Martins D. (2020). ISA 100.11a Networked Control System: Evaluating Link Stability for Robust Wireless Control. Sensors.

[10] Hasegawa T., Yamamoto S. Design and execution of a ’Plant Wide ISA100 Wireless’ network for optimization of complex process industries. Proceedings of the 2015 54th Annual Conference of the Society of Instrument and Control Engineers of Japan (SICE).

[11] (2020). IEEE Standard for Low-Rate Wireless Networks—Part 15.4: Wireless Medium Access Control (MAC) and Physical Layer (PHY) Specifications for Low-Rate Wireless Personal Area Networks (LR-WPANs).

[12] Sánchez-Sutil F., Cano-Ortega A. (2021). Smart Control and Energy Efficiency in Irrigation Systems Using LoRaWAN. Sensors.

[13] Emerson Automation Solutions (2020). Industrial Wireless Sensors: Use Cases for WirelessHART®® and LoRaWAN™ Protocols.

[14] Verma P., Shukla A.K., Kaur S. Arduino Sigfox in Smart Trash Can System. Proceedings of the 2021 9th International Conference on Reliability, Infocom Technologies and Optimization (Trends and Future Directions) (ICRITO).

[15] McClelland C. IoT Connectivity-Comparing NB-IoT, LTE-M, LoRa, SigFox, and other LPWAN Technologies. https://www.iotforall.com/iot-connectivity-comparison-lora-sigfox-rpma-lpwan-technologies.

[16] Phoenix Contact (2016). Simple But Reliable: Wireless Platform Improves Oil Field Notizfication System.

[17] Lethaby N. (2017). Wireless Connectivity for the Internet of Things: One Size Does Not Fit All.

[18] Emerson Process Management (2006). Plant Web University-Wireless 105: Wireless Topologies.

[19] Roshan P., Leary J. (2004). 802.11 Wireless LAN Fundamentals.

[20] Löser K. (2020). Wi-Fi 6 in the Industry.

[21] Gast M. (2005). 802.11 Wireless Networks: The Definitive Guide.

[22] Debnath S.K. (2018). Throughput Estimation Models under Various Conditions and MIMO Host Location Optimization Approach for Wireless Local-Area Network. Ph.D. Thesis.

[23] Aronoff H. WiFi Standards Explained: WiFi 4 vs WiFi 5 vs. WiFi 6. https://www.minim.com/blog/wifi-4-vs-wifi-5-vs-wifi-6.

[24] Alicia WiFi 6 Speed Explained: Boosting Your Home Network. https://reolink.com/blog/wifi-6-speed/.

[25] Wi-Fi Alliance Security. https://www.wi-fi.org/discover-wi-fi/security.

[26] Hakemeyer F. (2017). Trusted Wireless 2.0–Basics and Practical Applications.

[27] Field Comm Wirelesshart Security. https://www.fieldcommgroup.org/wirelesshart-security.

[28] Arbelaez A., Mehta D., O’Sullivan B., Quesada L. (2018). A Constraint-Based Parallel Local Search for the Edge-Disjoint Rooted Distance-Constrained Minimum Spanning Tree Problem. J. Heuristics.

[29] Butler J., Pietrosemoli E., Zennaro M., Fonda C., Okay S., Aichele C., Buettrich S., Forster J., Wierenga K., Vyncke E. (2013). Wireless Networking in the Developing World.

[30] Kuphaldt T.R. (2022). Lessons In Industrial Instrumentation.

[31] Reach G., Measurements Gridless Reach Website. https://www.gridlessreach.net/measurements.

[32] Phoenix Contact (2016). Real-Life Applications of Trusted Wireless.

[33] The Things Network The Things Network. LoRaWAN Radio Planning: Simulating your network deployment. Proceedings of the Things Conference.

[34] Huerta M.K., Garizurieta J., González R., Infante L.-Á., Horna M., Rivera R., Clotet R. (2023). A Long-Distance WiFi Network as a Tool to Promote Social Inclusion in Southern Veracruz, Mexico. Sustainability.

[35] Loeser K. IWLAN–Automating Together (iPCF). https://blog.siemens.com/2019/09/iwlan-automating-together-ipcf/.

[36] Phoenix Contact RAD-2400-IFS-Wireless Module–2901541. https://www.phoenixcontact.com/en-gb/products/wireless-module-rad-2400-ifs-2901541.

